# Sall4 Is Transiently Expressed in the Caudal Wolffian Duct and the Ureteric Bud, but Dispensable for Kidney Development

**DOI:** 10.1371/journal.pone.0068508

**Published:** 2013-06-18

**Authors:** Daichi Toyoda, Atsuhiro Taguchi, Masahiko Chiga, Tomoko Ohmori, Ryuichi Nishinakamura

**Affiliations:** 1 Department of Kidney Development, Institute of Molecular Embryology and Genetics, Kumamoto University, Kumamoto, Japan; 2 Japan Science Promotion Agency, CREST, Kumamoto University, Kumamoto, Japan; Leibniz Institute for Age Research - Fritz Lipmann Institute (FLI), Germany

## Abstract

The kidney, the metanephros, is formed by reciprocal interactions between the metanephric mesenchyme and the ureteric bud, the latter of which is derived from the Wolffian duct that elongates in the rostral-to-caudal direction. *Sall1* expressed in the metanephric mesenchyme is essential for ureteric bud attraction in kidney development. *Sall4*, another member of the *Sall* gene family, is required for maintenance of embryonic stem cells and establishment of induced pluripotent stem cells, and is thus considered to be one of the stemness genes. *Sall4* is also a causative gene for Okihiro syndrome and is essential for the formation of many organs in both humans and mice. However, its expression and role in kidney development remain unknown, despite the essential role of *Sall1* in the metanephric mesenchyme. Here, we report that mouse Sall4 is expressed transiently in the Wolffian duct-derived lineage, and is nearly complementary to Sall1 expression. While Sall4 expression is excluded from the Wolffian duct at embryonic (E) day 9.5, Sall4 is expressed in the Wolffian duct weakly in the mesonephric region at E10.5 and more abundantly in the caudal metanephric region where ureteric budding occurs. Sall4 expression is highest at E11.5 in the Wolffian duct and ureteric bud, but disappears by E13.5. We further demonstrate that *Sall4* deletion in the Wolffian duct and ureteric bud does not cause any apparent kidney phenotypes. Therefore, Sall4 is expressed transiently in the caudal Wolffian duct and the ureteric bud, but is dispensable for kidney development in mice.

## Introduction

The mammalian kidney, the metanephros, is formed by reciprocally inductive interactions between two precursor tissues, the metanephric mesenchyme and the ureteric bud [1]. The mesenchyme attracts the ureteric bud, while the ureteric bud induces the mesenchyme to differentiate into the epithelia of the glomeruli and renal tubules. Meanwhile, the ureteric buds branch and differentiate into collecting ducts and the ureter. The ureteric bud is derived from the Wolffian duct (nephric duct) that elongates in the rostral-to-caudal direction and reaches the caudal end of the embryo at embryonic (E) day 9.5. At E10.5, the Wolffian duct stimulates the nephrogenic mesenchyme to form mesonephric tubules [2]. The caudal end of the Wolffian duct adjacent to the metanephric mesenchyme bulges, from which ureteric budding occurs at E11.0. Subsequently, the ureteric bud elongates and starts to branch. The ureteric buds branch repetitively until a few days after birth in mice.

*spalt* (*sal*) was first isolated from *Drosophila* as a region-specific homeotic gene and encodes a nuclear protein characterized by multiple double zinc finger motifs [3]. The *Sall* (sal-like) family is conserved among species, and humans and mice each have four *sal-like* genes (known as *SALL1–4* in humans and *Sall1–4* in mice). Mutations in *SALL1* and *SALL4* have been associated with Townes-Brocks syndrome and Okihiro syndrome, respectively, which are both autosomal dominant diseases that involve abnormalities in various organs, including the ears, limbs, heart, and kidneys [4,5]. Okihiro syndrome is likely to result from *Sall4* haploinsufficiency, because *Sall4* heterozygous mice exhibit similar phenotypes to the human symptoms [6]. *Sall4* is essential for the maintenance of embryonic stem cells [6], and accumulating evidence indicates that *Sall4* is involved in the pluripotency network in stem cells [7–9]. *Sall4* is also involved in the establishment of induced pluripotent stem cells, and is therefore considered to be one of the stemness genes [10,11] In contrast, *Sall1* is expressed in the metanephric mesenchyme of the embryonic kidney, and *Sall1* knockout mice die shortly after birth with kidney agenesis [12]. In *Sall1*-null mice, the ureteric bud fails to invade the metanephric mesenchyme at E11.5, meaning that *Sall1* is required for this key step of metanephros development. The other phenotypes observed in Townes-Brocks syndrome are not apparent, because truncated Sall1 proteins produced in humans are likely to inhibit other Sall family proteins in a dominant-negative manner [13]. Indeed, *Sall1* cooperates with *Sall3* and *Sall4* in the formation of multiple organs, such as the limb buds, heart, and anus, as revealed by compound mutants [6,14]. However, the role of *Sall4* and its relationship with *Sall1* in kidney development remain unclear.

To examine the roles played by *Sall4* in kidney development, we analyzed Sall4 expression during kidney development. We found that Sall4 is transiently expressed in the ureteric bud lineage, which is distinct from the expression patterns of Sall1.

## Materials and Methods

### *Ethics statement*

All animal experiments were approved by the Animal Care and Use Committee of Kumamoto University (# A24-110). Protocols were performed in accordance with the NIH Guide for the Care and Use of Laboratory Animals. The mice were euthanized by cervical dislocation performed by well-trained individuals.

### *Generation of mutant mice*

*Hoxb7GFP*, *Hoxb7Cre*, and *Sall4* ^*flox/flox*^ mice were described previously [9,15,16]. *Hoxb7GFP* and *Hoxb7Cre* mice were obtained from the Jackson Laboratory. Mice carrying the Cre allele were genotyped with forward primer Cre 1 (5’-AGGTTCGTTCACTCATGGA-3’) and reverse primer Cre 2 (5’-TCGACCAGTTTAGTTACCC-3’), which give a 250-bp product. The Sall4 allele was genotyped with the following primers: 5’-CCTCCCGGAATTGCTTATCT-3’, 5’-AGGACAAGGATCGTTCTACAGC-3’, 5’-CTGTCCATCTGCACGAGACT-3’ (wild-type allele: 178 bp; floxed allele: 400 bp). Polymerase chain reaction (PCR) amplifications were performed under identical conditions using GoTaq DNA polymerase (Promega), with denaturation at 95°C for 5 min, 35 cycles of 95°C for 30 s, 58°C for 60 s, and 72°C for 30 s, and a final extension at 72°C for 7 min. The PCR products were analyzed by electrophoresis in a 1.2% agarose gel and visualized by ethidium bromide staining.

### *Immunostaining*

Histologic examinations were performed as described previously [17,18]. Mice were fixed in 10% formalin, embedded in paraffin, and cut into 6-µm sections. Immunostaining was performed using a BlueMap Kit (Roche) and an automated Discovery System (Roche) according to the manufacturer’s protocols. The following primary antibodies were used: anti-Sall4 ( [6]; Perseus Proteomics); anti-Sall1 ( [19]; Perseus Proteomics); anti-GFP (Abcam); anti-cytokeratin (Sigma); and anti-Six2 (Proteintech).

## Results

### *Sall4 is not expressed in the Wolffian duct or nephrogenic mesenchyme at E9.5*.

**Figure 1:**
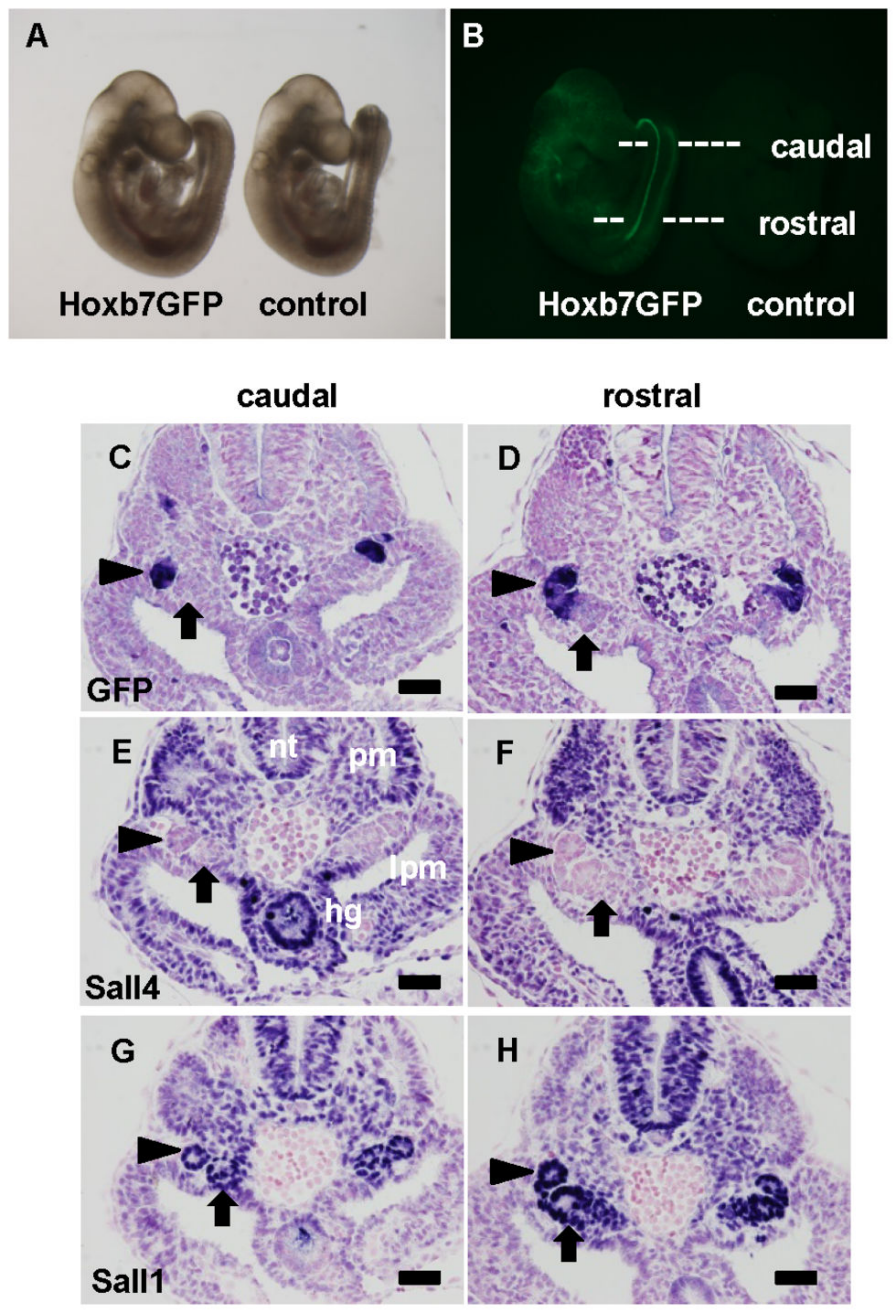
Sall4 is not expressed in the Wolffian duct or nephrogenic mesenchyme at E9.5. (A, B) Bright-field and fluorescence views of *Hoxb7GFP* and control mice at E9.5. The dotted lines indicate the planes used for the caudal and rostral sections in the following panels. (C, D) Green fluorescent protein staining of caudal and rostral sections from a *Hoxb7GFP* mouse. (E, F) Sall4 staining of the neighboring sections. (G, H) Sall1 staining of the neighboring sections. Arrowhead: Wolffian duct; arrow: nephrogenic mesenchyme; hg: hindgut; lpm: lateral plate mesoderm; nt: neural tube; pm: paraxial mesoderm. Scale bars: 50 µm.

First, we visualized the Wolffian duct using a transgenic mouse strain, in which green fluorescent protein (GFP) is driven by the *Hoxb7* promoter (*Hoxb7GFP*) [15]. We were able to observe the Wolffian ducts, which run in the rostral-to-caudal direction, in the lower half of the embryos at E9.5 (Figure 1A, B). In sections, GFP-positive Wolffian ducts were detected adjacent to the nephrogenic mesenchyme in both the caudal and rostral parts (Figure 1C, D). The ducts in the rostral part were more convoluted, because the differentiation of the Wolffian ducts proceeds at an earlier stage in the rostral part. Sall4 expression was undetectable in the Wolffian ducts and the nephrogenic mesenchyme, while its expression was detected in other areas, including the hindgut, lateral plate mesoderm, paraxial mesoderm, and neural tubes (Figure 1E, F). In contrast, Sall1 was expressed in both the Wolffian ducts and the nephrogenic mesenchyme, as well as in the lateral plate mesoderm, paraxial mesoderm, and neural tubes (Figure 1G, H). Therefore, Sall4 expression is excluded from the Wolffian duct and the nephrogenic mesenchyme at E9.5. Instead, Sall1 is expressed in these populations.

### *Sall4 is expressed in the caudal Wolffian duct at E10.5*

**Figure 2:**
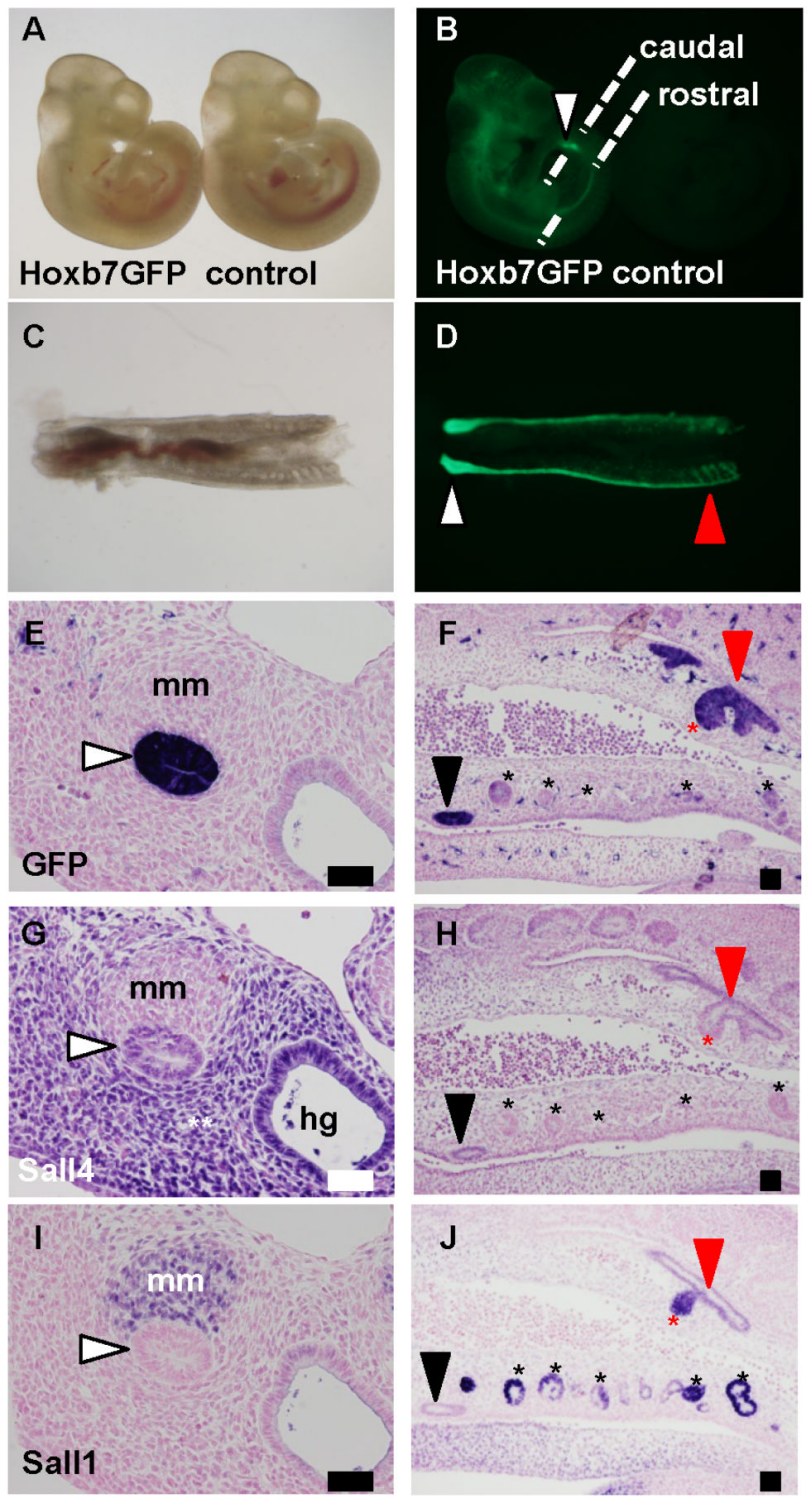
Sall4 is expressed in the caudal Wolffian duct at E10.5. (A, B) Bright-field and fluorescence views of *Hoxb7GFP* and control mice at E10.5. The dotted lines indicate the planes used for the caudal and rostral sections. (C, D) Bright-field and fluorescence views of the nephric region of a *Hoxb7GFP* mouse. (E, F) Green fluorescent protein staining of caudal and rostral sections from a *Hoxb7GFP* mouse. The right side of panel F is the rostral side. (G, H) Sall4 staining of the neighboring sections. (I, J) Sall1 staining of the neighboring sections. White arrowhead: caudal end of the Wolffian duct; black arrowhead: middle part of the Wolffian duct; red arrowhead: rostral part of the Wolffian duct; white double asterisk: tissues surrounding kidney primordia and hindgut; black asterisk: mesonephric tubule; red asterisk: rostral mesonephric tubule; hg: hindgut; mm: metanephric mesenchyme. Scale bars: 50 µm.

In *Hoxb7GFP* mice at E10.5, GFP-positive Wolffian ducts were visible in the lower half of the body, although GFP was also detected in the upper part of the body (Figure 2A, B). The strong signal detected close to the hindlimbs (Figure 2B, white arrowhead) corresponded to the caudal end of the Wolffian duct, which was more clearly demonstrated in the dissected urogenital regions (Figure 2C, D). This portion appeared to bulge, because the ureteric bud emerges at this region soon after this stage (Figure 2D, white arrowhead). In the rostral end, several branches were derived from the Wolffian ducts (Figure 2D, red arrowhead). In sections, the GFP-positive Wolffian duct was detected in the caudal end and adjacent to the metanephric mesenchyme (Figure 2E). In the middle and rostral parts, GFP was detected along the Wolffian duct in the mesonephric region (Figure 2F). In the most rostral end, branching of the Wolffian duct was confirmed, with the formation of rostral mesonephric tubules. However, the GFP staining of most of the mesonephric tubules located in the middle part was close to the background level, consistent with the notion that they are derived from the nephrogenic mesenchyme, and not from the Wolffian ducts.

At this stage, Sall4 was expressed in the Wolffian duct adjacent to the metanephric mesenchyme (Figure 2G), and more weakly expressed in the ducts in both the middle and rostral parts of the mesonephros (Figure 2H). Sall4 expression was also detected in the hindgut epithelia and the tissues surrounding the kidney primordia and hindgut. The metanephric mesenchyme, as well as most of the mesonephric tubules, was negative for Sall4. In contrast, Sall1 was expressed in these mesenchyme-associated regions, namely the metanephric mesenchyme and mesonephric tubules (Figure 2I, J). Weak Sall1 expression was also detected in the rostral part of the Wolffian duct. The rostral branch of the Wolffian duct, which was forming rostral mesonephric tubules, was also positive for Sall1. Therefore, the expressions of Sall4 and Sall1 are almost reciprocal. Sall4 is expressed in the Wolffian duct, more abundantly in the caudal end (metanephric region), while Sall1 is mainly expressed in the mesenchyme-associated tissues. Their expressions only overlap in the Wolffian ducts in the mesonephric region, but their expression levels are weak.

### *Sall4 is transiently expressed in the ureteric bud from E11.5 to E12.5*.

**Figure 3:**
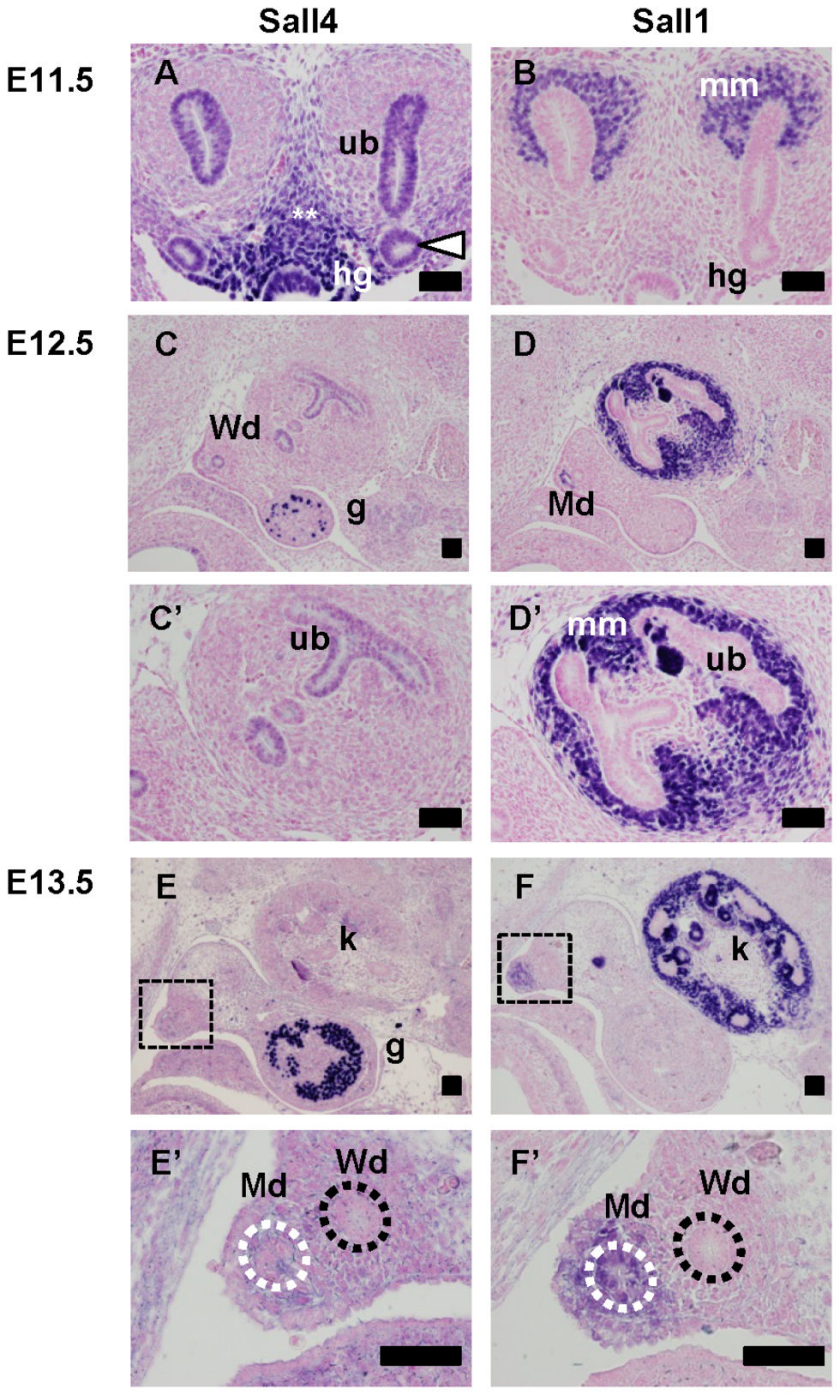
Sall4 is transiently expressed in the ureteric bud from E11.5 to E12.5. (A, B) Sall4 and Sall1 staining of E11.5 kidneys. (C, D) Sall4 and Sall1 staining of E12.5 urogenital regions. (C’, D’) Higher magnification of the kidneys in panels C and D, respectively. (E, F) Sall4 and Sall1 staining of E13.5 urogenital regions. (E’, F’) Higher magnification of the squares in panels E and F, respectively. White arrowhead: caudal end of the Wolffian duct; white double asterisk: tissues surrounding kidney primordia and hindgut; white dotted line: Müllerian duct; black dotted line: Wolffian duct; g: gonad; hg: hindgut; k: kidney; Md: Müllerian duct; mm: metanephric mesenchyme; ub: ureteric bud; Wd: Wolffian duct. Scale bars: 50 µm.

The ureteric bud branches out from the caudal part of the Wolffian duct at E11.5. Sall4 expression in the kidney was highest at this stage. Sall4 was expressed in both the Wolffian duct and ureteric bud, and in both the ureteric tip and stalk (Figure 3A). It was also expressed in the hindgut and surrounding tissues, similar to the findings for E10.5. In contrast, Sall1 was only expressed in the metanephric mesenchyme (Figure 3B). At E12.5 when the ureteric bud undergoes a few rounds of branching, Sall4 remained expressed in the ureteric bud epithelia, although its expression level was reduced (Figure 3C, 3C’). Sall4 expression was also detected in the residual Wolffian duct, and more abundantly in germ cells in the gonad. Sall1 expression was excluded from these regions, and detected in the metanephric mesenchyme surrounding the ureteric buds (Figure 3D, 3D’). At E13.5, Sall4 expression in the kidney and Wolffian ducts became undetectable, while strong expression in the gonads remained (Figure 3E, E’). In contrast, Sall1 was maintained in the metanephric mesenchyme (Figure 3F). Sall1 was also detected in the Müllerian duct epithelia and surrounding mesenchyme, which were newly formed adjacent to the Wolffian duct (Figure 3D, 3F, 3F’). Taken together, the expressions of Sall1 and Sall4 are mutually exclusive. Sall4 is transiently expressed from E10.5 to E12.5 in the Wolffian duct-derived epithelia, especially in the caudal Wolffian duct and the ureteric buds at the initial phase of budding and branching.

### *Sall4 expressed in the Wolffian duct and ureteric bud is dispensable for kidney development*

**Figure 4:**
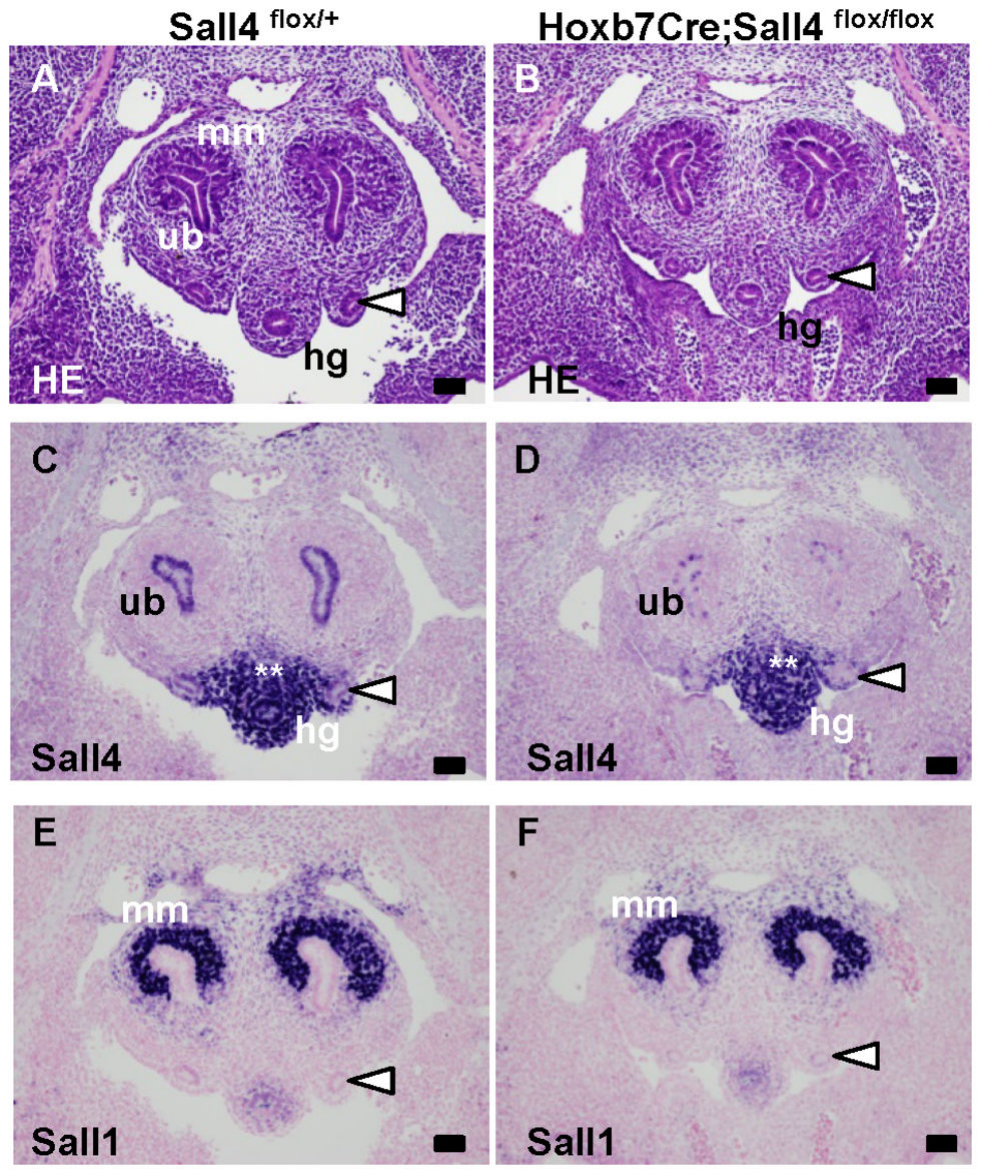
*Sall4* is deleted in the ureteric bud and Wolffian duct in *Hoxb7Cre; Sall4* ^*flox/flox*^ mice. (A, B) Hematoxylin and eosin staining of control and *Sall4* mutant kidneys at E11.5. (C, D) Sall4 staining of the neighboring sections. (E, F) Sall1 staining of the neighboring sections. White arrowhead: caudal end of the Wolffian duct; white double asterisk: tissues surrounding kidney primordia and hindgut; hg: hindgut; mm: metanephric mesenchyme; ub: ureteric bud. Scale bars: 50 µm.

To address the roles of Sall4 in the Wolffian duct and ureteric bud, we crossed mice carrying the floxed allele of *Sall4* with *Hoxb7Cre* mice, which expressed Cre recombinase in these lineages. When analyzed at E11.5, the ureteric bud was formed in *Sall4* mutant mice (Figure 4A, B), and Sall4 expression in the ureteric bud and Wolffian duct, but not in areas surrounding the hindgut, was significantly reduced in the *Hoxb7Cre;Sall4* ^*flox/flox*^ embryos (Figure 4C, D). These findings indicate successful and specific deletion of the gene, although a small number of Sall4-expressing cells remained through incomplete *Cre*-mediated excision. Sall1 expression in the metanephric mesenchyme was unaffected in the absence of *Sall4* (Figure 4E, F).

**Figure 5:**
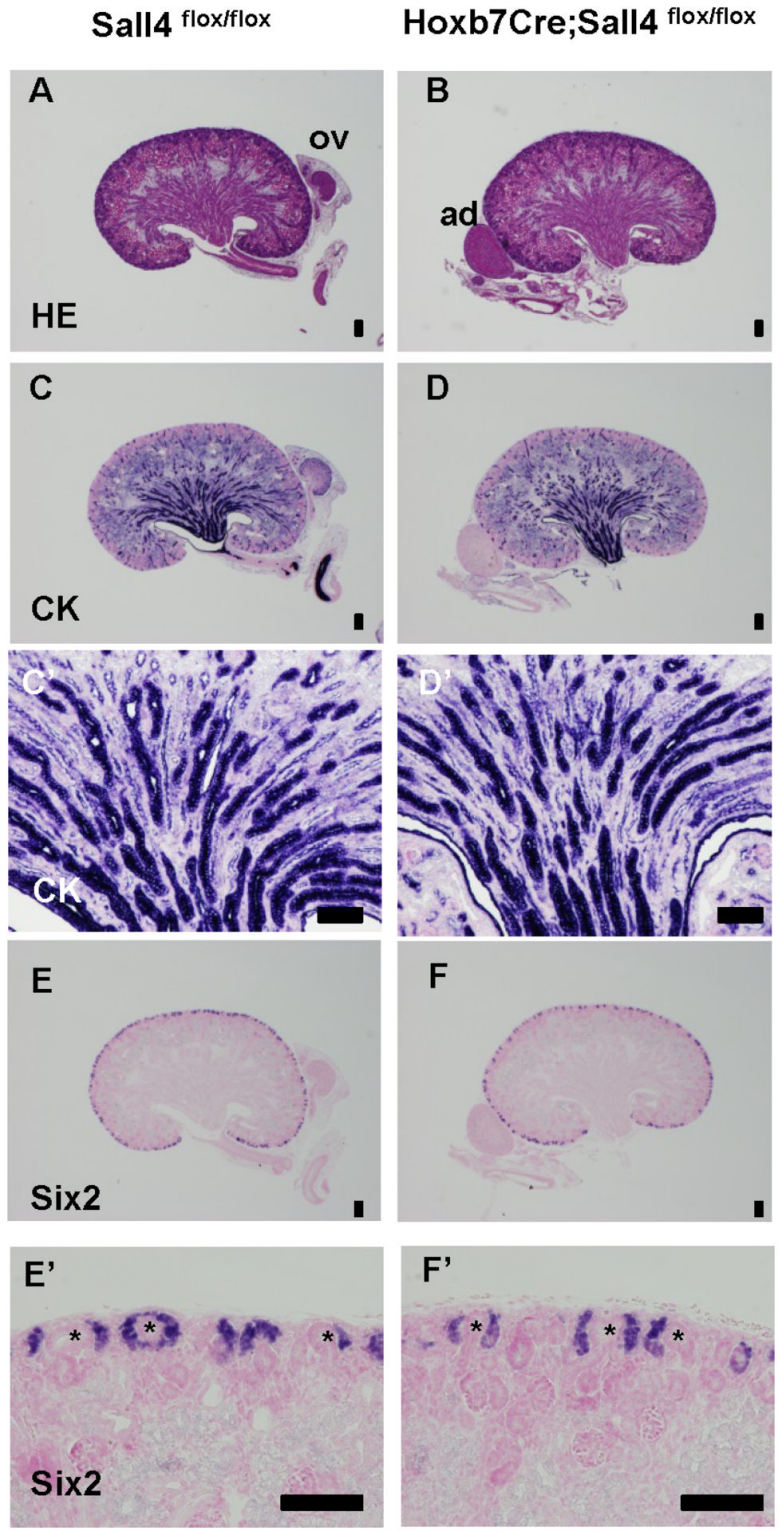
Sall4 expressed in the Wolffian duct and ureteric bud is dispensable for kidney development. (A, B) Hematoxylin and eosin staining of control and *Sall4* mutant kidneys in newborn mice. (C, D) Cytokeratin staining of the neighboring sections. (C’, D’) Higher magnification of the kidneys in panels C and D, respectively. (E, F) Six2 staining of the neighboring sections. (E’, F’) Higher magnification of the cortical areas in panels E and F, respectively. Asterisk: ureteric bud; ad: adrenal gland; ov: ovary. Scale bars: 100 µm.

*Hoxb7Cre;Sall4* ^*flox/flox*^ mice were born without any apparent phenotypes and survived until adulthood (n=3). In newborns, the sizes and structures of the mutant kidneys were indistinguishable from those in control mice (n=3; Figure 5A, B). Staining for cytokeratin, which marks the ureteric bud-derived structures, showed that the kidney development was largely unaffected (Figure 5C, C’, D, D’). At this stage, the metanephric mesenchyme, which is positive for the transcription factor Six2, surrounded each ureteric bud tip at the periphery of the kidney (Figure 5E, E’). Therefore, Six2 staining also served as an indirect assay to examine the development of the ureteric bud in the cortex. In the mutant kidneys, we were also able to observe the Six2-positive mesenchyme around the ureteric bud tips, and there were no apparent differences from the control kidneys (Figure 5F, F’). Therefore, Sall4 expressed in the Wolffian duct and ureteric bud is dispensable for their development during kidney formation.

## Discussion

We have shown that Sall4 expression in the kidney is almost complementary to that of Sall1. Sall1 is expressed in the mesenchyme-associated lineages, namely the metanephric mesenchyme and mesonephric tubules, the latter of which are derived from the nephrogenic mesenchyme. In contrast, Sall4 is expressed in the Wolffian duct and its derivative, the ureteric bud. Sall4 expression is more abundant in the caudal Wolffian duct just before the ureteric bud is formed and is retained in the ureteric bud that arises from the Wolffian duct. However, Sall4 expression is only retained in the initial phase of ureteric branching, and Sall4 is thus transiently expressed from E10.5 to E12.5 in the developing kidney.

The ureteric bud undergoes repetitive branching until birth, which is mainly regulated by mesenchyme-derived Gdnf and its receptor Ret in the ureteric bud tips. Since these molecules remain expressed from the initial phase of branching until birth, the branching forefront of the ureteric buds could be genetically identical throughout the process. However, our data indicate that the Sall4-positive cells, which are transiently found at the initial phase of the ureteric budding and branching, are likely to be distinct from the ureteric buds formed later in development. Since *Sall4* is one of the stemness genes, it is attractive to consider that Sall4 also marks the immature ureteric bud population. Indeed, it has been proposed that β-catenin, as well as *Gata3*, keeps the cells of the ureteric bud and Wolffian duct in a precursor state [20,21]. It would be interesting to examine Sall4 expression under *Hoxb7Cre*-mediated deletion or overexpression of β-catenin or *Gata3*. Our data also suggest that Sall4 could be used as a marker in attempts to induce immature ureteric buds from stem cells, although careful consideration should be paid because Sall4 is expressed in various extrarenal tissues.

Sall4 expressed in the Wolffian duct and ureteric bud is dispensable for kidney development. Since Sall1 is not expressed in the caudal Wolffian duct at E10.5 or in the ureteric buds at any stages, Sall1 could have no overlapping roles with Sall4 in the ureteric budding. Therefore, deletion of both *Sall1* and *Sall4* using *Hoxb7Cre* would be unlikely to lead to severe kidney phenotypes. Although antibodies against Sall2 and Sall3 applicable for immunostaining are not available, previous reports using *in situ* hybridization indicate that *Sall2* and *Sall3* are both mainly expressed in the metanephric mesenchyme [22,23]. Therefore, *Sall4* could be the only *Sall* family gene expressed in the ureteric bud, but is not required for its development. On the other hand, some patients suffering from Okihiro syndrome, which is caused by *SALL4* mutations, exhibit kidney abnormalities [5]. In addition, some Sall1/Sall4 compound heterozygous mutant mice show kidney agenesis or hypoplasia [6]. Therefore, a cell population should exist that shows coexpression of Sall1 and Sall4 and is essential for kidney development. One possibility is the Wolffian ducts in the mesonephric region at E10.5, but they expressed Sall1 and Sall4 only weakly. Another population may exist at earlier stages.

In addition to the kidney, Sall4 is expressed in a variety of tissues, including the heart, ears, limb buds, anus, and germ cells in the gonads [6,24]. In this study, we have further added its expression in the hindgut, lateral plate mesoderm, paraxial mesoderm, and neural tubes. Analyses of the roles of Sall4 in these tissues should lead to elucidation of the common and different molecular events regulating organ development.
